# Improving postoperative radiographs for the parameter measurement of hexapod external fixator using an additional foot ring

**DOI:** 10.1186/s13018-021-02820-9

**Published:** 2021-11-13

**Authors:** Yanshi Liu, Kai Liu, Feiyu Cai, Tao Zhang, Aihemaitijiang Yusufu

**Affiliations:** 1 Department of Trauma and Microreconstructive Surgery, The First Affiliated Hospital of Xinjiang Medical University, Urumqi, Xinjiang China; 2 Department of Orthopedics and Trauma, Tianjin Hospital, Tianjin, China

**Keywords:** Deformity measurement, Hexapod external fixator, Limb rotation, Orthogonality, Postoperative radiographs

## Abstract

**Background:**

It is challenging to determine the orthogonality of radiographs in daily clinical practice. The purpose of this study was to show the usefulness of an additional foot ring which might determine the orthogonality of postoperative radiographs for the parameter measurement of hexapod external fixator.

**Methods:**

We retrospectively analyzed 81 consecutive trauma patients with tibial shaft fractures treated by the hexapod external fixator at our institution from September 2014 to July 2019. Starting in March 2016, the postoperative radiographs for parameter measurement were obtained under the control of an additional foot ring. The final data consisted of 47 patients in traditional radiographs (Group I) and 34 patients under the control of foot ring during the radiographic process (Group II). The demographic data, original postoperative deformities, residual deformities after final correction, number of repeated radiographs after the first postoperative radiographs, time to the satisfactory reduction achieved, and external fixation time in all patients were documented and analyzed. The Johner–Wruhs criteria were used for the final clinical outcomes evaluation at the last clinical visit.

**Results:**

Satisfactory reduction and bone union were achieved in all patients. There were no statistical significances between the two groups in the demographic data, original postoperative deformities, residual deformities after final correction, external fixation time, and the final clinical outcomes (*P* > 0.05). The mean number of repeated radiographs after the first radiographs (1.4 times) and mean time to the satisfactory reduction achieved (3.3 days) in patients with an additional foot ring used were all less than those without foot ring (2.4 times, 5.3 days) (*P* < 0.05).

**Conclusions:**

The additional foot ring is a practical device to ensure the orthogonality of postoperative radiographs for the hexapod external fixator parameter measurement. Radiation exposure, duration of deformity correction, and cost for patients might be reduced due to the less repeated radiographs with the wrong position.

## Background

The circular external fixators are equipped with the ability to eliminate bending and translational shear while maintaining a degree of axial micromotion [1–3], providing a three-dimensional stable biomechanical environment that is conducive to bone healing and regenerate formation [4]. Hexapod external fixation (HEF) systems, such as the Taylor spatial frame (TSF), are a modification of the traditional Ilizarov circular external fixator [5]. The HEF is comprised of two rings connected by six telescopic struts, imparting the frame with six-degrees-of-freedom. This arrangement enables one ring can be multidimensionally repositioned with respect to the other one by adjusting strut lengths, therefore, allowing simultaneous correction of spatial deformities assisted by a specific software without frame alternation. As the expertise of this versatile device was gained by more general orthopedic surgeons, the HEF is increasingly used for trauma-control, posttraumatic reconstruction, and deformities correction [6–12].

For deformity correction and fracture reduction using the HEF, stable fixation that translates all movement from the rings directly to the corresponding bony segments was needed firstly, and followed by accurate radiographs analysis for deformity correction planning. Parameters regarding the bony deformities and how the frame is mounted are all required to be measured on the postoperative two-dimensional orthogonal radiographs [13–15]. The long-leg radiograph is the gold standard for measuring limb alignment and planning the deformity correction in the coronal plane [16]. Therefore, to take the standard anteroposterior (AP) X-rays, the patella should be orientated precisely to the center of the femoral condyles due to the limb is neutrally rotated when the patella pointing forward, the feet should point forward at the same time [15]. However, it is challenging to determine the orthogonality of AP and lateral X-rays in daily clinical practice [17], especially in patients with polytrauma and severe deformities. Many previous published data have reported that radiographs performed with extremity malrotation will lead to wrong measurements of the mechanical axis [18–20], while it is difficult to assess this malrotation. These inaccurate radiographs always result in a time-consuming correction process due to the unsatisfactory results are often required repeated radiographs, exposing the patient to further radiation exposure.

In our institution, a simple device was used to control the limb position and allows the radiographer to make the two radiographs adequately orthogonal to each other. The device can be easily installed and is suitable for all surgeons working with a hexapod system. The purpose of this study was to show the usefulness of this device which might determine the orthogonality of radiographs.

## Methods

This study retrospectively analyzed 81 trauma patients with tibial shaft fractures treated by the hexapod external fixator (Tianjin Xinzhong Medical Instrument Co., Ltd., Tianjin, China) at our institution from September 2014 to July 2019, including 68 males and 13 females with an average age of 38 years (range 18–64 years). The hexapod external fixation treatments were conducted due to trauma-control and correction of multiplanar posttraumatic deformities with poor surrounding soft tissues that were inadvisable for conventional internal fixation. Postoperative deformities greater than 5° or 10 mm in any anatomical plane were needed to take standard radiographs to plan fracture reduction [21].

**Fig. 1:**
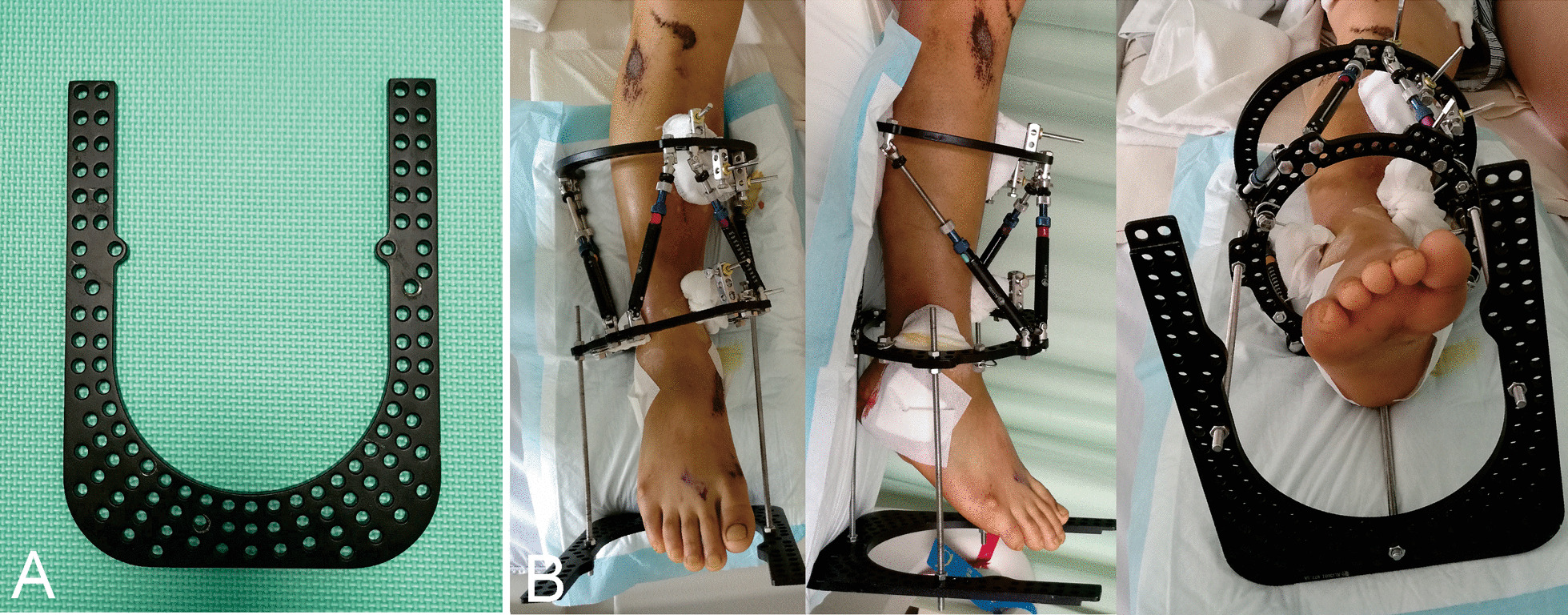
**A** General appearance of a foot ring. **B** Installation of the foot ring on the distal hexapod ring

Starting in March 2016, the postoperative radiographs for parameter measurement were obtained under the control of an additional foot ring (Tianjin Xinzhong Medical Instrument Co., Ltd., Tianjin, China) (Fig. 1). The final data consisted of 47 patients in traditional radiographs (Group I) and 34 patients under the control of foot ring during the radiographic process (Group II). The demographic data, original postoperative deformities, residual deformities after final correction, number of repeated radiographs after the first postoperative radiographs, time to the satisfactory reduction achieved, and external fixation time in all patients were retrospectively documented and analyzed. Informed consent was acquired from all patients for their information to be recorded and published in the present study. The Ethical Committee of our institution approved this study.

### Radiographs management

Parameters need to be measured on the postoperative X-rays include six deformity parameters and four mounting parameters. The deformity parameters include translation and angulation in coronal, sagittal, and axial plane respectively. The mounting parameters describe the location of the reference ring center relative to the origin point, including anteroposterior view frame offset, lateral view frame offset, axial view frame offset, and the rotary frame angle (defined as the rotation of the reference ring relative to the reference bony fragment). However, the rotational parameters in axial plane are traditionally determined by clinical examination due to the absence of axial spatial information on 2D radiographs [12].

All radiographs were justly taken for clinical reasons rather than the purpose of this study. The postoperative AP and lateral X-rays were conducted subjectively by radiologists in the traditional way without any accessory equipment before March 2016.

**Fig. 2:**
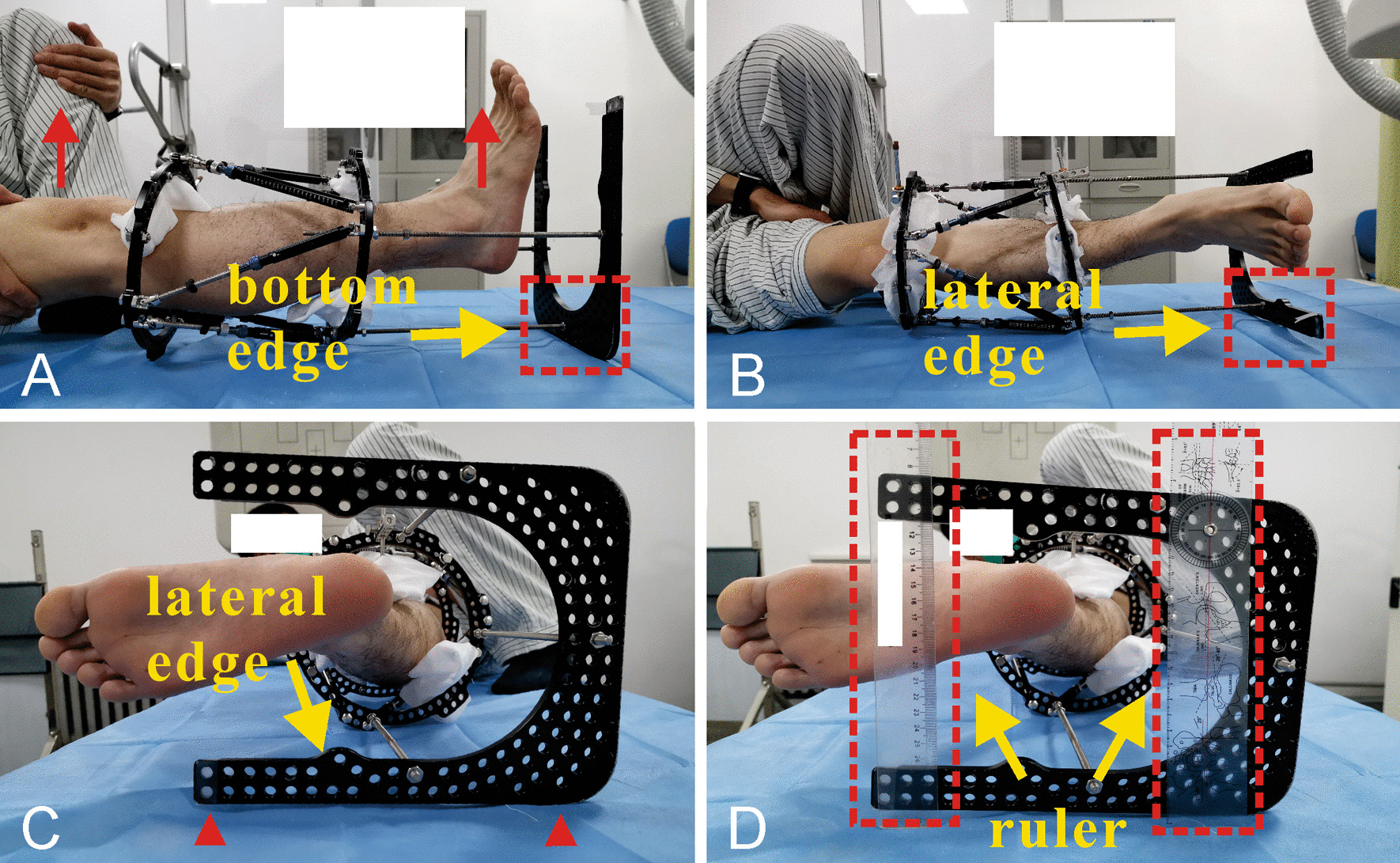
The schematic images of patient position when taking radiographs. **A** AP view: ensuring the lower leg was in a neutral position (the patella was orientated precisely to the center of the femoral condyles, and the feet should point forward at the same time) when the bottom edge of the foot ring was flat on the examining table or parallel to the horizontal line. **B** and **C** Lateral view: rotating the lower leg and ensuring the lateral edge of the foot ring are flat on the examining table or parallel to the horizontal line. **D** Two rulers were used to ensure the edge of the foot ring was parallel to the horizontal line when the edge could not flat on the examining table

As shown in Fig. 2, starting from March 2016, the postoperative radiographs were obtained under the control of an additional foot ring via the same radiographer and radiological machine. In the radiographic process, an additional foot ring was attached to the distal hexapod ring via three threaded rods. The additional foot ring was cyclically utilized when radiographs were taken each time. For the anteroposterior X-ray, adjusting the mounting holes on the distal hexapod ring and the foot ring to ensure that the lower leg was in a neutral position (the patella was orientated precisely to the center of the femoral condyles, and the feet should point forward at the same time [15]) when the bottom edge of the foot ring was flat on the examining table or parallel to the horizontal line (Fig. 2a). As for the lateral radiograph, rotating the lower leg ensures that the side perpendicular to the bottom edge (lateral edge) of the foot ring are flat on the examining table or parallel to the horizontal line (Fig. 2b, c). In addition, two rulers were usually used to ensure the edge of the foot ring was parallel to the horizontal line when the edge could not flat on the examining table (make sure that any two points on the edge of the foot ring are the same distance from the horizontal line) (Fig. 2d). In this simple way, the radiologist and the patient himself can easily control the rotation position of the limb while taking the radiographs, and the two X-rays are orthogonal to each other.

### Fracture reduction and effectiveness evaluation

Thirteen parameters needed by the computer program were calculated based on the postoperative AP and lateral X-rays. Deformity measurements of the injured limb on the radiographs were performed using CorelDRAW X7(Corel, Canada) with an accuracy of 0.01 mm. The residual deformities were evaluated by the same observer who is experienced in musculoskeletal radiology.

All patients underwent the total residual program of the HEF. Fracture reduction was performed by gradual strut adjustment according to the electronic prescription. The rate of strut adjustment was modified according to patients’ tolerance. If a satisfactory reduction has not been achieved, repeated radiographs were taken to continue the reduction planning. After the final correction, the reduction effectiveness was evaluated by the translation and angulation in the AP and lateral view according to the standard orthogonal radiographs (the patella was orientated precisely to the center of the femoral condyles and the feet was pointed forward at the same time in the AP view).

The hexapod external fixation was removed when sufficient union (corticalization in 3 of 4 cortices) was shown. All patients were followed up at a minimum of 12 months after the fixator removal. The final clinical outcomes were evaluated by the Johner–Wruhs criteria [22] at the last clinical visit.

### Statistical analysis

Statistical analysis was performed with the SPSS 22.0(IBM Corp, USA). Distribution of the data were evaluated by Kolmogorov–Smirnov test and Shapiro–Wilk test. Continuous variables were analyzed by Independent-samples T-tests or Mann–Whitney U test, expressing as the mean ± standard deviation and range of the observations. The count variables were analyzed by the Chi-square or Fisher’s test, representing as a number. A statistically significant difference was set at *P* < 0.05.

## Results

**Fig. 3:**
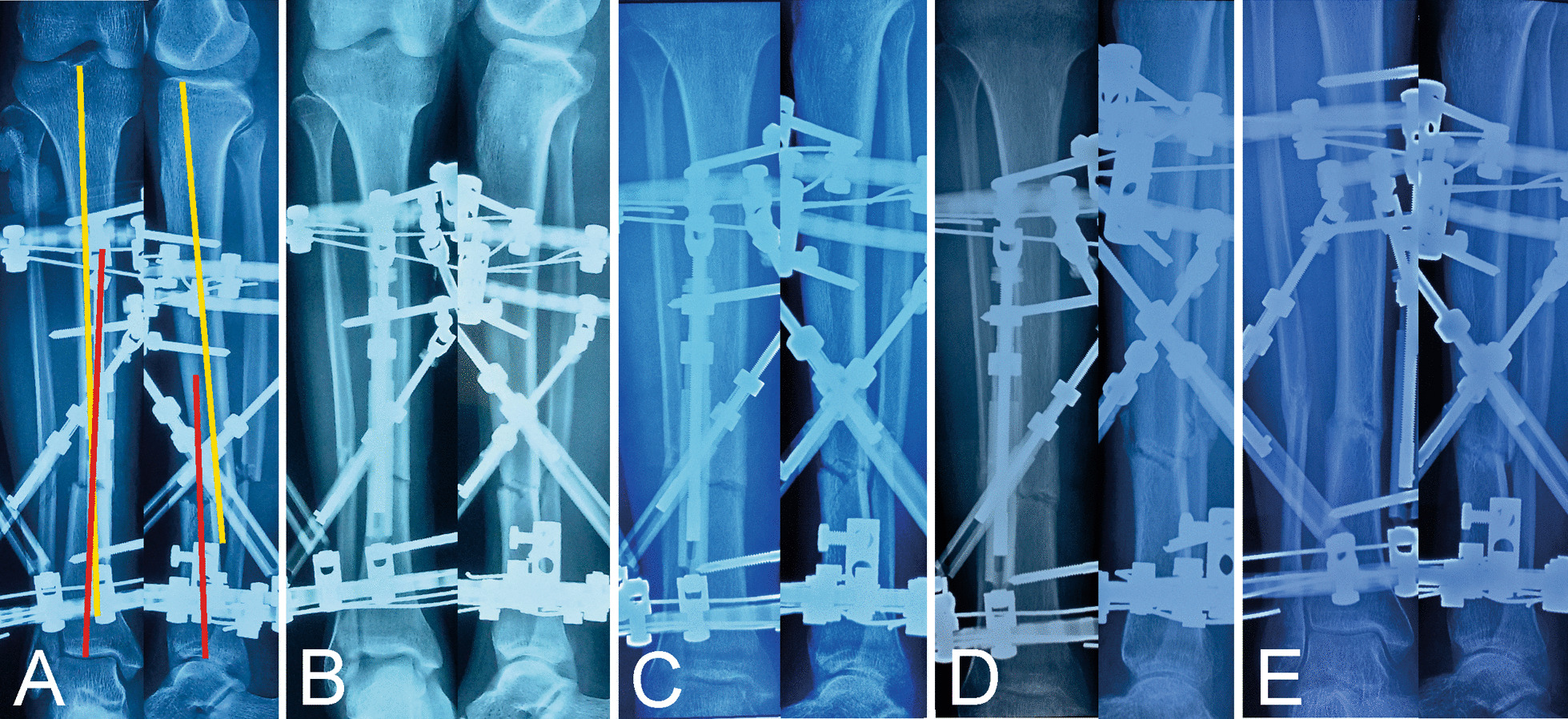
Images of a 37-year-old man with posttraumatic multidimensional deformities in tibia treated by the hexapod external fixator. **A** Radiographs immediately after installation of HEF. **B** Radiographs immediately after final correction. **C** Radiographs one month later. **D** Radiographs three months later. **E** Radiographs five months later

**Fig. 4:**
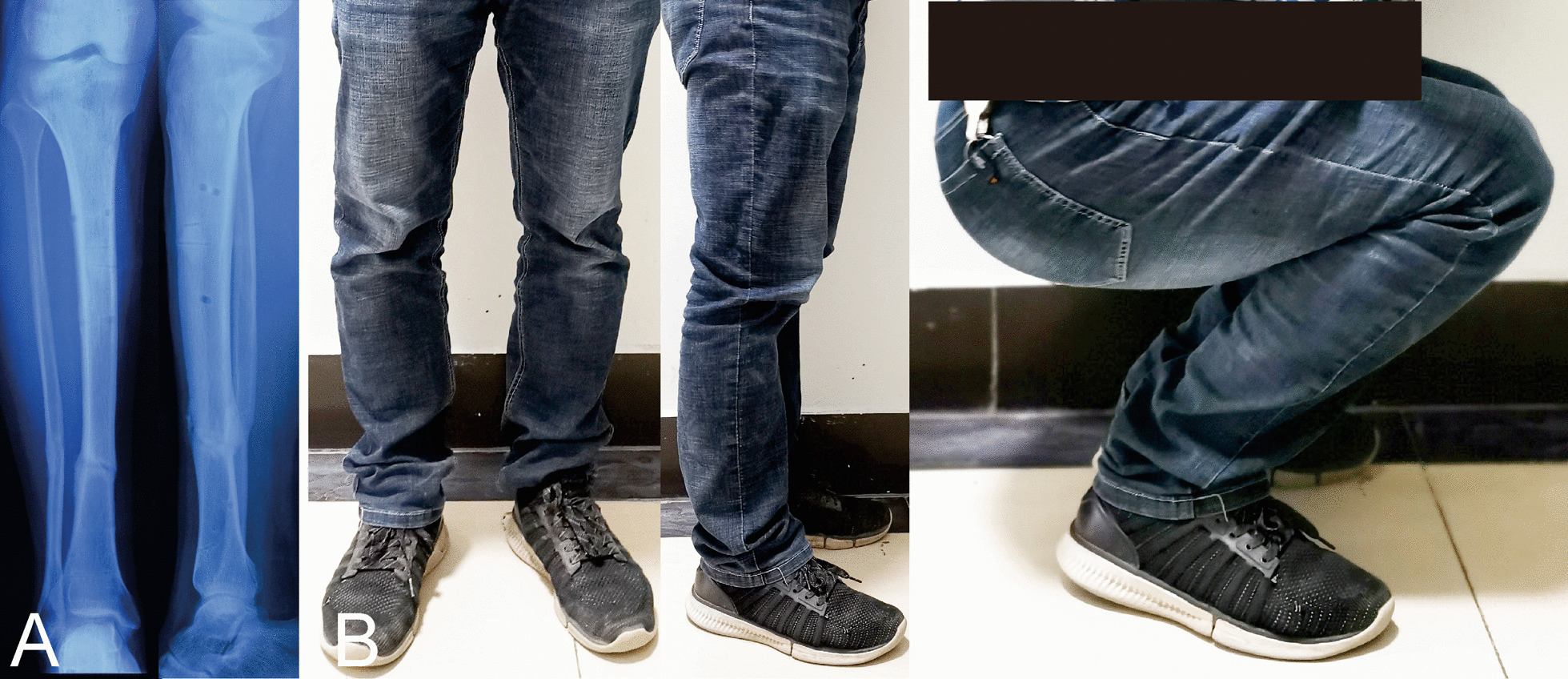
Follow-up images of the same patient after removing the HEF. **A** Radiographs six months later. **B** Clinical images of the patient, obtained at 12 months after HEF removal

Satisfactory reduction and bone union were achieved in all patients. The mean follow-up after HEF removal was 16.0 months (range 12–26 months), and no patient was lost to follow up, as well as no refracture was observed. (Typical case was shown in Figs. 3, 4).

For the demographic data, original postoperative deformities, residual deformities after final correction, external fixation time, and the final clinical outcomes, there were no statistically significant differences between the two groups (*P* > 0.05). The mean number of repeated radiographs after the first radiographs (1.4 times) and mean time to the satisfactory reduction achieved (3.3 days) in patients with an additional foot ring used were all less than those without foot ring (2.4 times, 5.3 days). All the differences between the two groups were statistically significant (*P* < 0.05). (More details are shown in Tables 1 and 2).

**Table 1:** Overview of demographic data between the two groups

	Without foot ring	With foot ring	Statistical value	P value
*Patients*				
Male	39	29	0.078	0.779
Female	8	5		
Age (year)	38.3 ± 12.0 (18–64)	37.1 ± 9.8 (20–59)	− 0.216	0.829
*Injury mechanism*				
Road traffic accident	35	23	2.662	0.268
Fall from height	7	3		
Crushing injury	5	8		
*Open/closed fracture*				
Open	33	29	2.499	0.183
Closed	14	5		
*OTA classification of fractures*				
A	12	8	0.792	0.638
B	28	23		
C	7	3		
Time elapsed since the injury to HEF installation (day)	3.4 ± 1.5 (1–7)	3.2 ± 1.4 (1–6)	0.625	0.534
*Original postoperative deformities*				
T1 (mm)	6.6 ± 4.2 (0–15.7)	7.0 ± 4.4 (0–17.7)	− 0.316	0.752
A1 (°)	4.6 ± 2.3 (1.4–11.2)	4.8 ± 2.1 (0–8.7)	− 0.991	0.322
T2 (mm)	5.7 ± 4.1 (0–14.1)	6.4 ± 4.1 (0–15.4)	− 0.933	0.351
A2 (°)	4.2 ± 2.5 (0–11.3)	3.3 ± 2.3 (0–9.7)	− 1.676	0.094

T1: translation deformities in the coronal plane

A1: angulation deformities in the coronal plane

T2: translation deformities in the sagittal plane

A2: angulation deformities in the sagittal plane

**Table 2:** Clinical outcomes of the two groups

	Without foot ring	With foot ring	Statistical value	P value
*Residual deformities after final correction*				
T1 (mm)	2.2 ± 1.3(0–4.3)	1.8 ± 1.4(0–3.9)	− 1.199	0.230
A1 (°)	0.9 ± 0.6(0–1.8)	0.8 ± 0.6 (0–1.5)	− 0.431	0.667
T2 (mm)	1.4 ± 1.1 (0–3.2)	1.1 ± 0.9 (0–2.4)	− 1.107	0.268
A2 (°)	1.0 ± 0.8 (0–2.1)	0.8 ± 0.7 (0–2.2)	− 0.789	0.430
N (time)	2.4 ± 0.8 (1–4)	1.4 ± 0.5 (1–2)	6.674	*P* < 0.001
Time to satisfactory reduction achieved (day)	5.3 ± 2.1 (1–9)	3.3 ± 1.0 (1–5)	− 4.562	*P* < 0.001
External fixation time (week)	26.3 ± 5.1 (16–41)	26.8 ± 5.2 (17–40)	− 0.211	0.833
Follow-up (month)	15.8 ± 3.2 (12–25)	16.4 ± 3.4(12–26)	− 0.838	0.404
*Johner–Wruhs criteria*				
Excellent	35	28	0.800	0.760
Good	9	5		
Moderate	3	1		
Poor	0	0		

T1: Residual translation in the coronal plane

A1: Residual angulation in the coronal plane

T2: Residual translation in the sagittal plane

A2: Residual angulation in the sagittal plane

N: number of repeated radiographs after the first postoperative radiographs

## Discussion

The hexapod external fixator provides advantages of simultaneous correction of multiplanar spatial deformities without frame modification, playing a vital role in orthopedic and reconstructive surgery [6–12, 23]. Accurate radiographic analysis of bony deformities and mounting parameters are crucial for the success of hexapod external fixation treatment. Postoperative adjustments require precise radiographic imaging of the frames and fracture site in both the standard AP and lateral views. These radiographs must be taken in the orthogonal plane to generate accurate prescriptions based on a computer program [13, 14, 24]. However, it may be difficult to achieve in the common clinical practice. Many radiographs are usually obtained subjectively by radiographers, and they are not absolutely orthogonal for the postoperative deformities measurement. Inaccurate radiographic imaging can lead to wrong parameter measurements for hexapod external fixator, resulting in incorrect prescriptions as well as insufficient deformity correction [18–20, 25].

Lots of previously published methods have been described to obtain the standard orthogonal radiographs for postoperative correction planning of hexapod external fixator. Deakin et al. [14] used a frame-mounted spirit level to help the radiographer produce perfectly aligned radiographs. Ahrend et al. [15] conducted postoperative radiographs with the help of a rotation rod, concluding that the variability of rotation on radiographs was lower with the rotation rod and more reproducible and better comparable radiographs can be obtained. Kanellopoulos et al. [13] developed a noninvasive guiding frame to conduct reproducible and consistent x-rays oriented orthogonally to the reference ring at different points in the correction. Although satisfactory results of reducing repeated radiographs have been determined by the aforementioned techniques, it seems time-consuming to work in inexperienced hands.

Gantsoudes et al. [26] obtained intraoperative orthogonal images with the help of a rod marker, while these images were usually inadequate that just covered a small visual field, and the radiographic process might add anesthesia time. Besides, Sokucu et al. [27] declared that there is no difference between measurements taken during perioperative fluoroscopy and postoperative radiograph. Wright et al. [24] introduced a silhouette technique to obtain adequate orthogonal imaging, resulting in an improvement in the adequacy of planning imaging and a reduction of repeated radiographs requirement. Subsequently, Al-Uzri et al. [28] also designed and described a guideline to improve the quality of postoperative radiographs significantly. Compared to two-dimensional radiographs, the computed tomography remains the gold standard for accurate parameter measurement with the additional advantage of rotational deformities calculation [29]. However, there is a drawback of significantly higher radiation exposure.

In the present study, a noninvasive and simple device was used to improve postoperative radiographs for the correction planning of hexapod external fixator. Basic principles of imaging via orthogonal views were used in this technique. In fact, even if the anteroposterior X-rays is not taken in the neutral position of the limb, as long as the anteroposterior and lateral X-rays are taken when the two perpendicular sides of the foot rings are parallel to the horizontal line, the two radiographs taken at this time are perpendicular to each other. In the two consecutive groups, there were no statistically significant differences in the demographic data, original postoperative deformities, residual deformities after final correction, external fixation time, and the final clinical outcomes. However, compared to patients without additional foot ring used, there were fewer repeated radiographs after the first postoperative radiographs and less mean time to the satisfactory reduction achieved in patients with additional foot ring used. Our results manifested this device may ensure orthogonal radiographs for the parameter measurement, resulting in less radiation exposure and correction duration.

The additional foot ring is a user-friendly and cost-efficient device. It is easy for both the patient and radiographer to control the limb rotation and determine the radiographs' orthogonality during the radiographic process, just making the two perpendicular sides of the foot ring parallel to the horizontal plane respectively. Notably, the foot ring can be reused without increasing the cost burden on patients. Furthermore, radiation exposure, duration of deformity correction, and cost for patients might be reduced due to the less repeated radiographs with the wrong position.

According to our experience, this device has demonstrated an improvement in the orthogonality of postoperative radiographs for hexapod external fixator and a reduction in repeated imaging requirements. The radiographers involved also conclude that this way can easily obtain a good orthogonal view. Although we do not accurately define the radiation exposure for repeat imaging, radiation exposure reduction can be extrapolated due to the fewer repeated images.

The present study had several limitations. First of all, considering the small sample size, a conservative attitude should be adopted regarding the interpretations of our results. Besides, the patient has to be turned in an inconvenient position, especially for those with polytrauma or severe limb deformity, and it may be considered one limitation of this study. Moreover, during the anteroposterior view, adjusting the mounting holes on the distal hexapod ring and the foot ring to ensure that the lower leg was neutral may also be a time-consuming process, and an installation-friendly device is therefore needed to resolve this problem. Finally, if there was any rotational correction, it will change the position of ankle and distal bony end, and repeated mounting parameters measurement is needed.

## Conclusion

A significant improvement in the postoperative radiographs has been achieved in this study. The additional foot ring is a practical device to ensure the orthogonality of postoperative radiographs for the hexapod external fixator parameter measurement. Radiation exposure, duration of deformity correction, and cost for patients might be reduced due to the less repeated radiographs with the wrong position.

## Data Availability

The datasets analysed during the current study are available from the corresponding author on reasonable request.

## Abbreviations

HEF: Hexapod external fixator
TSF: Taylor spatial frame
AP: Anteroposterior
